# Multisite hernia treatment: the robotic approach makes it feasible

**DOI:** 10.3389/fsurg.2025.1711703

**Published:** 2025-11-05

**Authors:** Eva Carmen Diaz Casanova, Francesco Mongelli, Sebastiano Spampatti, Davide La Regina, Fabio Garofalo, Fabiano Iaquinandi, Ramon Pini, Johannes Maria Alberto Toti

**Affiliations:** 1 Department of Surgery, Bellinzona e Valli Regional Hospital, EOC, Bellinzona, Switzerland; 2 Faculty of Biomedical Sciences, Università Della Svizzera Italiana, Lugano, Switzerland

**Keywords:** multisite hernia, multiquadrant hernia, combined hernias, robotics abdominal wall surgery, docking technique

## Abstract

**Background:**

The use of robotic surgery for combined abdominal wall hernias, including multiquadrant hernias, is underexplored in the literature. While the prevalence of simultaneous hernias is not well documented, they represent a frequent clinical challenge.

**Aims:**

This study aimed to evaluate the feasibility of a robotic approach for treating simultaneous epigastric, umbilical, incisional, and inguinal hernias.

**Materials and Methods:**

We retrospectively reviewed a prospectively maintained dataset of abdominal wall hernias to identify patients treated for combined hernias (i.e., incisional/umbilical/epigastric and inguinal). Patients were divided into two groups based on the robotic docking technique, and the data were analyzed.

**Results:**

From January 2020 to December 2024, 30 patients underwent robotic combined hernia repair. Ninety percent were male, with a median age of 64.0 years (56.3–73.3). Most patients (56.7%) had an ASA score of 2. Single docking was feasible for 9 of 30 patients with midline hernias with median diameter of 2.0 cm (1.6–3.0) combined with an unilateral inguinal hernia. Double docking was necessary for 70% of patients with wider midline hernia defect with median diameter of 3.0 cm (2.0–5.0) or bilateral inguinal hernias. No intraoperative complications or conversions were reported. The median operative time was 158.0 min (141.0–160.0) for the single docking and 238.0 min (178.0–268.8) for the double docking and the median hospital stay was 2.0 days (2.0–2.0) for the single docking and 3.0 days (2.0–3.0) for the double docking. The morbidity rate was 11.1% for the single docking and 23.8% for the double docking, only one reintervention was needed in the double docking group. Most of the complications in both groups were seromas or hematomas, managed conservatively. At a median follow-up of 15.6 months (6.6–30.4), no recurrences were observed.

**Conclusions:**

Robotic combined hernia repair is a safe and effective minimally invasive option. Single docking offers advantages but is limited to patients with midline defects combined with unilateral inguinal hernias. For midline defects combined with bilateral inguinal hernias, double docking is generally required.

## Introduction

The surgical treatment of combined abdominal wall hernias, such as multisite hernias, remains underexplored, particularly with the use of robotic surgery. The multiquadrant robotic approach is gaining interest due to its ability to address multiple abdominal regions with a minimally invasive technique (1). However, this approach of reconstruction in simultaneous repair of epigastric, umbilical, incisional and inguinal hernias remains underdescribed in the literature (2, 3).

Abdominal wall hernias are common, affecting 4% of adults over 45 years old and 1.7% of the general population (4). Inguinal hernias account for over 75% of cases, while incisional hernias occur in 12.8% 2 years after a midline incision (5). Umbilical hernias are common and may be present in up to 25% of the population (6, 7). The incidence of combined hernias, involving multiple types in a single patient, remains poorly understood.

Currently, there are no large series or clear guidelines in the literature regarding the treatment of multisite combined hernias. The choice of surgical approach—open or laparoscopic—largely depends on the individual surgeon's experience, as evidence comparing thetechniques for this specific condition is lacking.

The evolution of hernia repair techniques has enabled a tailored approach for each patient. Surgeons can now address multiple defects, even in different anatomical sites, within a single surgical session. This strategy aims to reduce overall recovery time while ensuring effective repair.

This study aims to analyze the benefits of robotic surgery in the multiquadrant approach for the simultaneous repair of epigastric, umbilical, incisional, and inguinal mono and bilateral hernias, focusing on perioperative outcomes providing technical details to simplify combined robotic hernia repair.

## Material and methods

At our institution, which is a specialized referral center for minimally invasive abdominal wall surgery, we retrospectively searched from a prospectively maintained and audited database patients who underwent robotic-assisted surgery for multisite hernias (https://www.herniamed.de). The search was carried out from January 2020 to December 2024. We excluded patients operated with laparoscopy or open surgery and those not agreeing to participate to the study. Both primary and recurrent hernias were included. The primary endpoint was to assess safety and feasibility of the multisite hernia treatment as new concept of care for combined abdominal wall hernias.

We retrieved data on age, sex, height, weight, body mass index (BMI), presence of comorbidities (cardiac disease, arterial hypertension, smoking status, pulmonary disease, cirrhosis, renal disease, diabetes mellitus, anticoagulant medication), American Society of Anesthesia (ASA) score, previous abdominal surgery, type of surgical approach [i.e., extended totally extraperitoneal repair (eTEP) lateral or sovrapubic approach; extended transabdominal preperitoneal repair (eTAPP); single docking and double docking], type of hernia, hernia defect size and other hernia characteristics (location, primary, recurrent) according to EHS classification (8), type, dimension and number of implanted mesh, time of surgery, intraoperative complications and postoperative complications according to the Clavien-Dindo classification (9) and hospital length of stay (LOS). The follow-up included a clinical assessment 30 days after surgery and subsequently annually for up to five years post the hernia operation.

All operations were performed with the da Vinci Xi® platform. The surgical technique rTAPP for inguinal hernia and the eTEP approach for midline primary or incisional defects were already described in previous publications of our research group (10–13). In case of unilateral inguinal hernia combined with a midline primary or incisional hernia with small defect size (<4 cm) the lateral single docking technique was used, instead for the bilateral inguinal hernias and wider midline hernia defects the double docking technique was adopted.

The dissection plane is primarily preperitoneal whenever feasible, particularly for small to medium-sized primary defects. In contrast, a retromuscular approach is preferred for larger and incisional hernias. Each procedure is carefully tailored according to the defect characteristics, their anatomical location, and the combination of multiple defects. Furthermore, patient-specific factors such as prior surgical history, age, quality of life, and activity level play a critical role in surgical planning. Due to the complexity often associated with multiple hernias, advanced imaging studies are frequently recommended to enable precise preoperative assessment and optimal operative strategy.

The study was approved by the local ethic committee (Comitato Etico Cantonale Ticino, 2019–01132 CE 3495), and informed consent was obtained from included patients. This research was conducted in accordance with current international regulations. Strengthening the reporting of observational studies in epidemiology (STROBE) guidelines were followed (14).

## Statistical analysis

Descriptive statistics were presented as absolute frequencies for categorical variables and median with interquartile range (IQR) for continuous variables. All analyses were performed using MedCalc Statistical Software version 19.5.3 (MedCalc Software Ltd, Ostend, Belgium; https://www.medcalc.org; 2020).

## Results

During the study period, 30 patients who underwent multisite combined hernia repair were retrieved. Median age was 64.0 years (56.3–73.3), 27 patients were male (90%), median BMI was 26.8 kg/m^2^ (24.2–29.3) and most patients (56.7%) were classified ASA II.

The three most frequent comorbidities were arterial hypertension (33.3%), cardiac (26.7%) and pulmonary diseases (26.7%). Other comorbidities included diabetes mellitus (13.3%), smoking (13.3%), cirrhosis (6.7%) and renal disease (6.7%).

Concerning the hernia defect characteristics, the epigastric, umbilical and incisional hernias were grouped in W1 and W2–3 and in M1–5, according to the EHS Classification (8). The inguinal hernias were divided into unilateral and bilateral and, according to the EHS Classification, in lateral vs. medial and primary vs. recurrent hernias (15). The majority of hernias were primary defects in both groups, to be accurate, 66.7% of midline hernias and 95.1% of inguinal hernias. Specifically, 88.9% of midline hernias in the single docking and 57.1% in the double docking, 100% of inguinal hernias in the single docking and 93.8% in the double docking were primary defects. The midline hernia defects were divided in epigastric, umbilical and incisional hernias. The epigastric hernia covered in the single docking and in the double docking 11.1% and 9.5% respectively; umbilical hernias 77.8% and 47.6% respectively and incisional hernias 11.1% and 42.9% respectively. Hernia defect diameter of midline hernias was calculated.

Single docking was feasible for 9 of 30 patients with W1 midline hernias with median defect diameter of 2.0 cm (1.6–3.0) combined with an unilateral inguinal hernia. All epigastric (11.1%), umbilical (77.8%) and incisional (11.1%) hernias were W1 in the single docking and the majority were M3. The inguinal hernias in the single docking were all unilateral and primary hernias, of which 55.5% were medial hernias.

Double docking was necessary for 70% of patients with wider (W1–3) midline hernia defect, mean defect diameter of 3.0 cm (2.0–5.0) or bilateral inguinal hernias. The epigastric (9.5%), umbilical (47.6%) and incisional (42.9%) hernias in the double docking were mostly W1 (66.7%) and M3 (76.2%). The inguinal hernias in the double docking were mostly bilateral (52.4%), primary (93.8%) and lateral (53.1%) hernias.

We analyzed if patients underwent previous surgeries and the type of surgeries they experienced, 33.3% of patients in the single docking and 71.4% of patients in the double docking underwent open surgery and 33.3% in the single docking and 9.5% in the double docking experienced minimally invasive surgery. All details about patients demographics and preoperative parameters are reported in Table 1.

**Table 1:** General patient characteristics.

Docking technique	All		Single Docking		Double Docking	
	N:30		N:9		N:21	
Age, y (IQR)	64.0 (56.3–73.3)		71.0 (65.0–77.0)		62.0 (53.0–71.0)	
Males, *n* (%)	90		100		85.7	
BMI, median (IQR)	26.8 (24.2–29.3)		27.1 (26.5–29.7)		25.2 (23.9–29.0)	
Comorbidities						
Cardiac disease, *n* (%)	26.7		55.6		14.3	
Arterial hypertension, *n* (%)	33.3		55.6		23.8	
Smoke, *n* (%)	13.3		0		19.0	
Pulmonary disease, *n* (%)	26.7		22.2		28.6	
Cirrhosis, *n* (%)	6.7		11.1		4.8	
Renal disease, *n* (%)	6.7		11.1		4.8	
Diabetes mellitus, *n* (%)	13.3		22.2		9.5	
Anticoagulant medication, *n* (%)	10.0		11.1		9.5	
ASA 1 status, *n* (%)	10.0		11.1		9.5	
ASA 2 status, *n* (%)	56.7		44.4		61.9	
ASA 3 status, *n* (%)	33.3		44.4		28.6	
Hernia defect characteristics						
*Midline defect*						
Hernia defect diameter, cm (IQR)	3.0 (2.0–4.0)		2.0 (1.6–3.0)		3.0 (2.0–5.0)	
*EHS classification*	Primary	Incisional	Primary	Incisional	Primary	Incisional
M1, *n*	1	1	1	1	0	1
M2, *n*	1	2	0	0	1	2
M3, *n*	18	6	7	0	11	5
M4, *n*	0	1	0	0	0	1
W1, *n* (%)	76.7		100.0		66.7	
W ≥ 2, *n* (%)	23.3		0.0		14.3	
*Inguinal defect*						
Inguinal unilateral, *n* (%)	63.3		100.0		47.6	
Inguinal bilateral, *n* (%)	36.7		0.0		52.4	
*EHS classification*						
L:M, *n* (%)	51.2:48.8		44.4:55.6		53.1:46.9	
P:R, *n* (%)	95.1:4.9		100:0		93.8:6.3	
Previous surgery						
Open, *n* (%)	60		33.3		71.4	
Minimally invasive, *n* (%)	16.7		33.3		9.5	

BMI, body mass index; ASA, American society of anesthesiologists; EHS, European hernia society; M(1–5), midline hernia; W(1–3), width of hernia; M, medial hernia; L, lateral hernia; P, primary hernia; R, recurrent hernia.

Regarding the intraoperative results, no complications or conversions were reported. When analyzing the surgical technique, the eTEP approach was predominantly used, accounting for 25 cases (83.3%). Among these, 20 cases (80%) belonged to the double docking group. In contrast, the TAPP approach was performed in 5 cases (16.7%), with 4 cases (80%) allocated to the single docking group.

The median operative time was 158.0 min (141.0–160.0) for the single docking and 238.0 min (178.0–268.8) for the double docking. The median number of mesh used for each patient was 2.0 (2.0–2.0) in the single docking and 2.0 (2.0–3.0) in the double docking. The median area of the mesh number 1 was 225.0 cm^2^ (150.0–225.0) and of mesh number 2 was 225.0 cm^2^ (225.0–225.0) in the single docking, in the double docking the median area of mesh number 1 was 225.0 cm^2^ (225.0–500.0) and of mesh number 2 was 225.0 cm^2^ (225.0–225.0). Intraoperative parameters are summarized in Table 2.

**Table 2:** Intraoperative parameter.

Docking technique	All	Single Docking	Double Docking
Operating Time, min (IQR)	185.0 (153.3–252.3)	158.0 (141.0–160.0)	238.0 (178.0–268.8)
eTEP approach, *n* (%)	25 (83.3)	5 (20)	20 (80)
TAPP approach, *n* (%)	5 (16.7)	4 (80)	1 (20)
Number of mesh pro patient, median (IQR)	2.0 (2.0–2.8)	2.0 (2.0–2.0)	2.0 (2.0–3.0)
Area of mesh 1, cm^2^ (IQR)	225.0 (225.0–332.0)	225.0 (150.0–225.0)	225.0 (225.0–500.0)
Area of mesh 2, cm^2^ (IQR)	225.0 (225.0–225.0)	225.0 (225.0–225.0)	225.0 (225.0 -225.0)
Conversion, *n* (%)	0	0	0
Intraoperative complication, *n* (%)	0	0	0

min, minutes; cm, centimeters; TAPP, transabdominal preperitoneal; eTEP, extended totally extraperitoneal repair.

The morbidity rate was 11.1% for the single docking, all of which were classified Grade I-II according the Clavien-Dindo Classification and 23.8% for the double docking, only with one reintervention needed to treat a bleeding due to a preperitoneal hematoma in the double docking group, which was classified CD Grade ≥ IIIb. All except one of the complications in both groups were surgical site occurrences, 11.1% in the single docking and 19.0% in the double docking, managed conservatively. One patient had postoperative ileus CD Grade II. No surgical site infections occurred. As for urinary retention, no case was reported in neither of the groups. The median hospital stay was 2 days (2–2) for the single docking and 3 days (2–3) for the double docking.

After surgery patients were followed up at one month and then after one year. In all our series, at a median follow-up of 15.6 months (6.6–30.4), 12.1 months (1.4–17.7) for the single docking and 22.7 months (10.1–30.4) for the double docking, no recurrences were observed. Postoperative parameters and follow up details are described in Table 3.

**Table 3:** Postoperative parameter and follow-up.

Docking technique	All	Single Docking	Double Docking
Postoperative complications			
Grade I–II CD, *n* (%)	13.3	11.1	19.0
Grade III–IV CD, *n* (%)	3.3	0	4.7
SSO, *n* (%)	16.6	11.1	19.0
SSI, *n* (%)	0	0	0
Urinary retention, *n* (%)	0	0	0
LOS, days (IQR)	2 (2–3)	2 (2–2)	3 (2–3)
Recurrence, *n* (%)	0	0	0
Follow-up, months (IQR)	15.6 (6.6–30.4)	12.1 (1.4–17.7)	22.7 (10.1–30.4)

CD, Clavien-Dindo classification; SSO, surgical site occurrence; SSI, surgical site infection; LOS, length of stay.

## Discussion

In our experience, robotic multisite hernia repair can be a safe and effective minimally invasive option.

Despite the existing skepticism concerning its usefulness, robotic surgery is gaining interest as an alternative approach for hernia repair. Indeed, some arguments in favor of robots, such as the feasibility of suturing instead of tacking to fix the mesh or the better surgeon ergonomics, appear undebatable (16).

The robotic approach in the simultaneous repair of multiquadrant hernias remains underexplored in the literature. In a recent case series, Anoldo et al. described their dual docking technique for repair of simultaneous inguinal and umbilical hernia (2). So far, more authors have dealt with the multiquadrant robotic approach in the treatment of colorectal cancer (17–20). Thanks to its robotic arms technology and integrated table motion, the da Vinci Xi system (Intuitive Surgical, Sunnyvale, CA) empowers the surgeon to carry on multiquadrant procedures. Our study shows encouraging results in terms of feasibility and safety of the robotic approach in the simultaneous treatment of epigastric, umbilical, incisional and inguinal hernias.

From a technical point of view, we found that single docking is particularly feasible in small umbilical or incisional hernias combined with a unilateral inguinal hernia, while double docking is necessary for wider W1–3 midline defect, whether primary or incisional and in case of combination with bilateral inguinal hernias. A relevant difference was observed in the distribution of surgical approaches between the two groups, with the eTEP technique predominantly adopted in the double docking group and the TAPP approach more frequently used in the single docking group. This divergence may reflect underlying differences in case complexity or surgeon preference. However, the interpretation of this finding should be approached with caution due to the limited sample size.

Switching from single to double docking has a certain relevance: as advantages of the single docking technique, a short operative time and a reduced number of trocar accesses are to mention. Arguably, the latter might be associated with lesser postoperative pain, reduced SSO risk and better cosmetic results. Of course, the operative time is mainly affected by the type and number of hernias. In our series, both docking techniques showed the multiquadrant approach to be feasible and safe, with no reported intraoperative complications and/or conversions, only minor post-operative complications and no recurrences at a median follow up time of 15.6 months.

As the benefit assessment of an intervention should be related to the patient (21), we should evaluate the robotic multisite hernia repair compared with a defined alternative treatment. Despite the lack of control group, if we assume that combined abdominal wall hernias are commonly treated through multiple procedures and repeated hospital admissions, we can see the multiquadrant robotic approach as a potentially added value to the patient. In addition, minimizing the number of hospitalization should have benefits in terms of healthcare costs.

This study has many limitations. Besides the above-mentioned lack of control group, it is based on a single surgical group's experience and a small sample size. Last but not least, our results are the consequence of a 10-year-long robotic experience using standardized procedures. A proper learning curve should be taken into account when considering starting with robotic abdominal wall surgery.

Based on our results, we can conclude that patients with simultaneous abdominal wall hernias can safely undergo minimally invasive surgery using a robotic platform, achieving favorable intra- and post-operative outcomes. These outcomes include low postoperative morbidity, such as a short hospital stay, minimal to no surgical site complications or infections, and no intraoperative conversions or post-operative recurrences.

## Data Availability

The raw data supporting the conclusions of this article will be made available by the authors, without undue reservation.
